# Exploring how technology influences out‐of‐home participation for persons living with dementia or mild cognitive impairment: a scoping review

**DOI:** 10.1002/alz70861_108776

**Published:** 2025-12-23

**Authors:** Natalie L. Mason, Lena McNeill, Amy S. Hwang, Sareh Zarshenas, Katerina Bekas, Manroop Khroud, Camille Normandin, Thomas TANNOU, Rosalie H. Wang

**Affiliations:** ^1^ University of Toronto, Toronto, ON Canada; ^2^ Centre de recherche de l'Institut universitaire de gériatrie de Montréal, Montreal, QC Canada; ^3^ CRIUGM, CIUSSS Centre‐Sud‐de‐l’Île‐de‐Montréal, Montreal, QC Canada

## Abstract

**Background:**

Aging‐in‐place presents unique challenges for persons living with dementia or mild cognitive impairment (PLWD/MCI). Cognitive deficits and unaccommodating environments often reduce PLWD/MCI’s access to and participation in places outside the home (e.g., public, commercial, transportation), even though out‐of‐home (OoH) participation is vital to maintaining health, wellbeing, and social citizenship. Technology is increasingly embedded in everyday environments, but its role in supporting OoH participation for PLWD/MCI has not been systematically reviewed. Using the Canadian Model of Occupational Participation, this scoping review synthesizes personal (i.e., meaning, purpose, history, relationships) and contextual (micro, meso, macro‐system) factors that shape how PLWD/MCI use technology to access and sustain OoH participation.

**Method:**

This review applied the Joanna Briggs Institute methodology and guidance. MEDLINE, Scopus, CINAHL, PsycINFO, AgeLine, Embase, and ACM Digital Library were searched for articles addressing: 1) the design/development or use of technological products/services (e.g., “navigation tool,” “wayfinding”), 2) out‐of‐home accessibility or participation (e.g., “neighbourhood,” “community”), and 3) PLWD/MCI (e.g., “dementia,” “Alzheimer disease”). After deduplication, 35280 articles were screened by title/abstract, 691 articles were full‐text screened, and 78 articles were included for data extraction.

**Result:**

Preliminary analysis results underscore the interplay between personal goals/values, history, relationships, socio‐environmental and structural contexts. For PLWD/MCI, OoH participation is not only functional but also meaningful, connoting freedom, belonging, identity, and social roles. Technologies can enable or disable this participation, and alter these meanings (e.g., GPS tracker may sustain freedom but imply dependence). PLWD/MCI with meaningful OoH motivations, past digital literacy, and supportive social networks are best positioned to sustain OoH participation. At the macro level, low public awareness and inadequate digital accessibility policies often create structural barriers (e.g., replacing in‐person staff with digital payment machines).

**Conclusion:**

Technologies embedded into OoH experiences of PLWD/MCI can enable or disable their meaningful and sustained participation, depending on interrelated personal and contextual factors. Assumptions of cognitive normativity and digital literacy in the design of OoH environments and technologies should be redressed by more dementia‐inclusive policies and attitudes that regard access to OoH places as central to health, social participation, and citizenship.

